# Ergonomic Rationalization Sequence of Digital Lighting Design in the Working Environment

**DOI:** 10.3390/ijerph19127275

**Published:** 2022-06-14

**Authors:** Darina Duplakova, Jan Duplak, Rastislav Kascak

**Affiliations:** Faculty of Manufacturing Technologies with a Seat in Presov, Technical University of Kosice, Bayerova 1, 080 01 Presov, Slovakia; darina.duplakova@tuke.sk (D.D.); jan.duplak@tuke.sk (J.D.)

**Keywords:** lighting, working environment, rationalization sequence, digital ergonomic design

## Abstract

This paper describes the creation of a rationalization sequence for working with simulation models, and its subsequent verification in the redesigning of lighting in cooperation with digital enterprise tools. The rationalization sequence consists of 11 sequences, whose accuracy is subsequently verified on a practical example of the redesign of an assembly room of a production hall in the simulation tool Dialux Evo. In conjunction with the proposed procedure, a redesign of the assembly workplace located in the production hall for daylighting, artificial, and mixed lighting was created. The conclusion of the paper provides an overview of the benefits gained from the application of the work environment rationalization procedure.

## 1. Introduction

Nowadays, simulation tools form an integral part of all manufacturing, controlling, and auxiliary processes in industry. Recently, there has been a growth of simulation tools in the field of ergonomics. Simulation tools are used in the field of ergonomics to simulate, evaluate, or rationalize physical loads, physical factors of the working environment, or risks at the workplace.

In the field of the application of ergonomic simulation tools for the comprehensive assessment of the physical load of workers, there is an important article by Zhang et al. [1], who deals with the ergonomic rationalization of welders’ standing postures. The authors created six digital welders using Jack human software, on which they implemented basic ergonomic methods for physical load assessment and determined the most suitable posture–operating distance, operating height, and rotation angle of the welder’s neck, in order to improve welders’ operational posture and prevent fatigue and injury to the welder. The above-mentioned software solution was also used in the determination of prediction algorithms for the identification of work positions in the implementation of four manual tasks with a focus on joint angles, low back forces, and strength capabilities, by digitizing real participants [2]. In addition to the Jack simulation tool, the issue of assessing the physical load of workers has also been explored using other simulation tools, such as the DHM tool IPS IMMA software, through which it is possible to assess workplaces, specifically the time and ergonomic parameters [3]. This software can also be implemented in proactive ergonomics, in the design of test procedures for the interior of the vehicle design process [4]. The software can also be applied as a standard for the assessment of the well-being of workers via the RULA score, as described in [5]. The implementation of software solutions for ergonomic load assessment of workers was also discussed by a pair of authors, Grobelny and Michalski [6], who digitized the workplace and workers using Anthropos ErgoMAX software, to devise a workplace design methodology that prevents work-related musculoskeletal disorders (WMSDs). The use of simulation tools is appearing more and more often in the assessment of physical factors, in conjunction with the assessment of emerging risks in the workplace. Predicting and evaluating noise, as one of the major environmental factors, through the use of software support is a highly topical issue, which can be confirmed by the contributions in [7,8,9,10], which deal primarily with mapping, simulation, and assessment of the impact of noise on workers. In addition to assessing noise as a physical factor in the work environment, simulation tools are used to assess thermal well-being at workplaces, as reported in [11,12,13,14]. The use of the most modern available tools of the digital age has created space for the gradual implementation of virtual reality in the conditions of ergonomic rationalization and assessment, of both the physical load of workers and the working environment as a whole. Regarding this issue, it is necessary to highlight, for example, the implementation of virtual reality as a supporting ergonomic tool for posture assessment in digital human modelling [15,16,17,18], or also as an ergonomic support tool in workplace design [19], or ergonomic risk assessment [20,21].

Currently, simulation tools are often used for modelling and evaluating the physical factors of the working environment, specifically for lighting modelling. Various companies are developing and improving simulation tools that provide information about the current state of lighting, but also about its rationalized or optimized state [22,23,24,25,26].

Despite the increasing use of simulation tools for the modelling and evaluation of lighting, there are not many publications on the subject at present. In 2015, Acosta et al. analyzed the precision of several lighting simulation programs regularly used in daylighting studies for architecture, following the methodology established in the CIE (The International Commission on Illumination) test cases document [27]. Simulation tools to assess daylighting have been widespread in the construction sector since the 1970s. In the last decade, several authors have dealt with the lighting simulation tools issue [28,29,30,31], but the methodology of working with these tools was not addressed. In 2016, author Pawlak determined the performance accuracy of the simulation of escape route lighting installations, using the most popular programs supporting lighting design: DIALUX and RELUX, as well as possible errors associated with illuminance measurement of emergency escape lighting [32]. Numerous evaluations of the simulation models available have been published. These can be divided into two groups: comparisons based on replicating a built reality (scale models or reality), and comparisons in controlled laboratory settings [33,34,35]. Commercial lighting simulation software can also be used for the analysis and calculation of various variables. The authors Son et al. used the Relux 2013 (Relux Informatik AG, Münchenstein, Switzerland), AGi32 v2.04 (Lighting Analysts, Littleton, CO, USA), and Dialux 4.11 software (DIAL GmbH, Lüdenscheid, Germany) for the calculation of the UGR (unified glare rating) parameter [36]. The simulation software can be used for a quantitative analysis of annual energy-saving potential from daylighting in a building or study and the simulation of indirect lighting system installations. [37,38]. Several scientific studies [39,40] have shown that the design of lighting systems often involves competing criteria, in addition to occupant comfort; a decision support system integrating daylight and/or other techniques can help the designer in the definition of the optimal design strategies. This system is a very important part of the modern design realization stage, but it is necessary to involve the human factor in the process. Despite individual research regularly being carried out in practice, there is currently little emphasis on the development of a general procedure or guidance on the use of digital tools in the field. For this reason, a general rationalization algorithm for working with simulation models was constructed.

Within the concept of the presented article, it is also necessary to define ergonomic rationalization in relation to the issue of lighting the working environment. In the general context, ergonomic rationalization can be defined as an activity that is implemented in the existing system; its structure and behavior are known, while the parameters are sought in which the behavior of the system with set criteria is considered the most advantageous. The generally interpreted determination of ergonomic rationalization can be defined within the issue of digital lighting design, as the implementation of all ergonomic tasks in the existing workspace (work environment), with the basic specification defining its characteristics (general purpose of the workspace/environment, layout specification, lighting system) by projecting this workspace/environment into digital form, where the most suitable parameters are sought and the phases ensuring the creation of a functional and reliable lighting system according to predetermined criteria are described.

The simulation tool Dialux Evo, which is described in the first part of the paper, was chosen for the realization of the constructed algorithm. The simulation tool was selected by comparing the three available software programs. Individual software was compared in terms of several criteria—availability, price, compliance with the standard, complexity of the working environment, interpretation, export of achieved results, etc. An overall comparison is also given in a previous article [41]. The objective of this article is the presentation and description of an ergonomic rationalization sequence that uses the principles of digital lighting design, with verification of its functionality on a practical model example.

After a brief introduction to the issue and an overview of similar research, the second part of the article focuses on the presentation of mathematical expressions needed for the subsequent calculation of lighting. The body of the article is divided into two sections: “Rationalization sequence of work with simulation models”, which is focused on the description of the created rationalization sequence; and “Application of comprehensive ergonomic sequence in practice”, which provides a verification of the created model directly, in practice, in an assembly hall of the production company. The conclusion of the article contains a discussion of the achieved results and their comparison, with a subsequent description of the benefits of the solution for science and practice in the Section 6.

## 2. Materials and Methods

The value of the actual daylight factor changes during the use of an interior and can be deemed an operational value. When making a decision with respect to a lighting opening at the design stage, a value that accounts for all the effects identified before the construction is required. These requirements translate into the resulting daylight factor *D*, expressed in %. It is a value determined by calculation under certain simplified assumptions. It is determined as the sum of the following components [42]:

(1) $$D=D_{S}+D_{E}+D_{i}=100\cdot \left(\frac{E_{is}}{E_{eH}}+\frac{E_{ie}}{E_{eH}}+\frac{E_{ir}}{E_{eH}}\right)$$

*D_s_*—the sky component of the factor expressing the ratio of the part of the interior lighting *E_is_*, caused by the sky brightness, and the actual horizontal lighting of an unshaded outer horizontal plane.

The sky component expresses the ratio by which a point on the plane under consideration in the interior is illuminated from the surface source element induced by the sky brightness, with respect to the current exterior horizontal lighting from an evenly clouded sky. The sky component may be obtained from the following relation [42]:

(2) $$D_{S}=\frac{\int L_{dS}\cos\vartheta \frac{\cos\psi }{l^{2}}dS}{E_{eH}}\cdot 100$$

*ψ*—angle of incidence of light rays on the lighting opening.
*ϑ*—angle of incidence of light rays on the point under consideration perpendicular to the plane of incidence,
*L_dS_*—luminance of the sky element with elevation angle *ε* = 90° − *ϑ*, calculated as follows [42]:

(3) $$L_{dS}=L_{Z}\frac{1+2\cos\vartheta }{3}\tau_{0}0,5\cos\psi \left(3-cos^{2}\psi \right)$$

since the horizontal exterior lighting is a function of the zenith brightness *Lz*, it can be defined by the following relation [42]:

(4) $$E_{eH}=\frac{7}{9}\pi L_{Z}$$

and thus, the general mathematical model for the sky component has the final form of [42]:

(5) $$D_{S}=\tau_{0}\frac{100}{\pi }A_{1}A_{2}\int \left(B+\cos\vartheta \right)\left(C-{cos}^{2}\psi \right)\cos\vartheta \frac{{cos}^{2}\psi }{l^{2}}dS$$

*A*_1_, *A*_2_, *B*, *C*—coefficients depending on the gradation of the sky brightness and the type of glazing.

*D_e_*—reflected external component of the factor expressing the ratio between the illuminated part *E_ie_* (light reflected from the reflective external surfaces, obstacles located in front of the building opening (windows) and the actual horizontal lighting. The mathematical formula is as follows [42]:

(6) $$D_{e}=\frac{D_{SF}}{10k_{\varepsilon }}$$

*D_i_*—reflected internal component of the factor expressing the ratio between the interior component of the light in the interior caused by the multiple reflection of daylight from the surfaces located in the interior of *E_ir_* and the actual horizontal lighting. The mathematical formula is as follows [42]:

(7) $$D_{i}=\frac{\chi \tau_{0}S_{0}}{S\left(1-\rho \right)}\left(D\rho_{d}+H_{\rho h}\right)$$

*χ*—correction factor,
*τ*_0_—light loss factor,
*S*_0_—convergence area of the lighting opening,
*S*—total area of internal surfaces,
*ρ*—average reflection factor of all internal surfaces,
*D*, *H*—constants,
*ρh_(ρd)_*—average reflection factor of the upper (lower) part of the room above the horizontal plane level.

The ergonomic rationalization sequence of the digital lighting design was conceived in accordance with lighting simulation tools that use the mathematical expressions described in above part of this article. All these mathematical expressions served as a supporting verification tool for the simulation data obtained.

## 3. Rationalization Sequence of Work with Simulation Models

**Figure 1:**
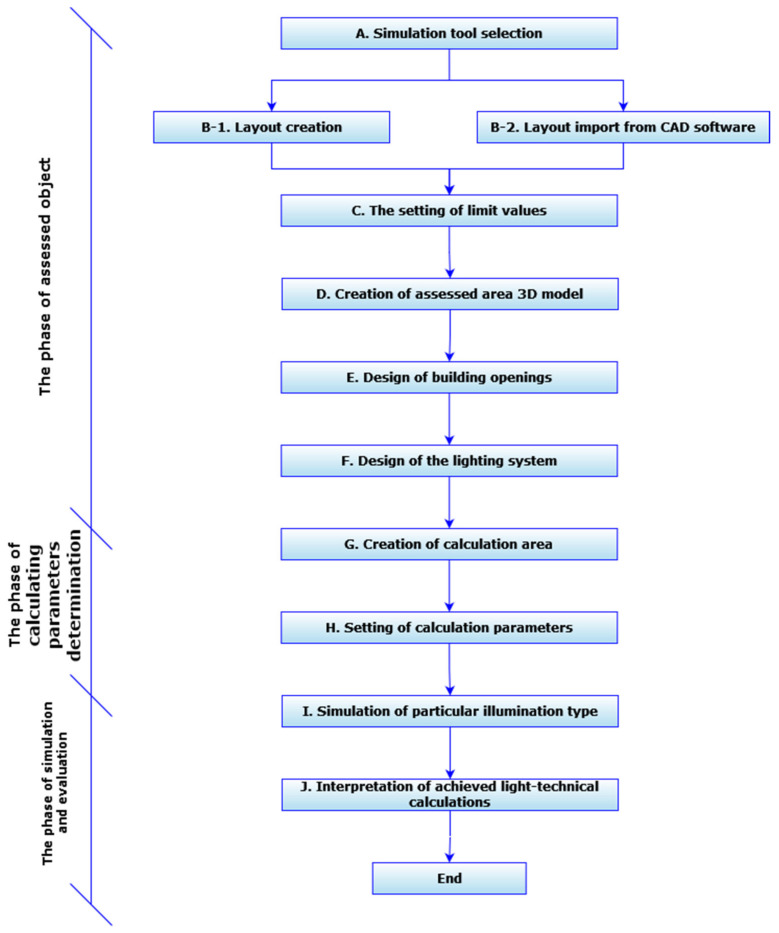
The procedure of work with simulation models.

During the creation of simulation models in lighting instruments, it is necessary to ensure the sequence of basic steps (A–J), which are shown and described in the following part of the work. This rationalization algorithm (Figure 1) forms a part of the main rationalization algorithm, which is presented and described in the first part of this paper: Ergonomic rationalization of lighting in the working environment, Part I [41].

The proposed sequence of the procedure is the following:

A. In the beginning, it is necessary to choose a suitable lighting simulation tool. The choice of simulation tool depends on various variables, such as its availability, price, and specification for a particular type of lighting.

B. In the second step, it is necessary to compose the current layout: the layout of the assessed object. There are two ways to build the layout: compiling in the CAD program, or in the light-technical program; with the possibility to export to a CAD program with extension dwg/dxf or import from CAD program with extension dwg/dxf.

C. The third step consists in setting the limit values for the assessment of the lighting by defining the type of the assessed object. This step is important in the final assessment of the suitability of the illumination, as the simulation tool, after defining these limit values that comply with EN 12 464-1 standard, can determine whether or not the achieved results meet or exceed the limit values.

D. After creating the layout and setting the limit values, it is possible to create a complex 3D model of the assessed space in the fourth step. To complete the model, it is necessary to define the height of the room and insert the required 3D objects, in accordance with the 2D layout.

E. The fifth step defines the basic building openings: windows, skylights, etc. This step is important during the identification of daylighting and mixed light. 

F. Calculating the artificial or mixed lighting is an important step. The sixth step defines the lighting system, by selecting artificial lighting from the manufacturer’s catalogues or importing from compatible files. This part completes the first phase: the construction phase of the assessed object.

G. and H. The second phase: the phase of defining the calculation parameters consists of two parts. In the first part, in the seventh methodological step, the computational surfaces (working plane) are created. Subsequently, in step number eight, the calculation parameters are set on these calculation surfaces as required. These parameters are set individually, to evaluate each type of lighting.

I. and J. In the last two steps, the third phase is applied: the simulation and evaluation phase. In this phase, the actual simulation of a specific type of lighting is realized and after its completion the achieved lighting–technical calculations are interpreted. Interpretation of results can be graphical or numerical, depending on the simulation tool used.

## 4. Application of a Comprehensive Ergonomic Sequence in Practice

As mentioned in the “Introduction” part of this article, verification of the rationalization procedure of working with simulation models is realized through the simulation tool Dialux Evo. A model of the daily, artificial, and associated lighting of an assembly room of 5875 × 6208 × 3000 mm was constructed for verification. The original layout of the room was proposed in 2015. In this proposal, it was planned to use this room as a production room, in which one lathe and one worktable were to be situated. Due to the fact that the company expanded during its existence, there has been a change that had to be addressed, to rationalize the original focus of the room. Before the rationalization, the room was a space without windows, with artificial lighting and individual devices. Considering the above, the general lighting was redesigned by means of simulation software, using a rationalization procedure, so that the room could be used for assembly purposes.

### 4.1. Daylighting Design

**Figure 2:**
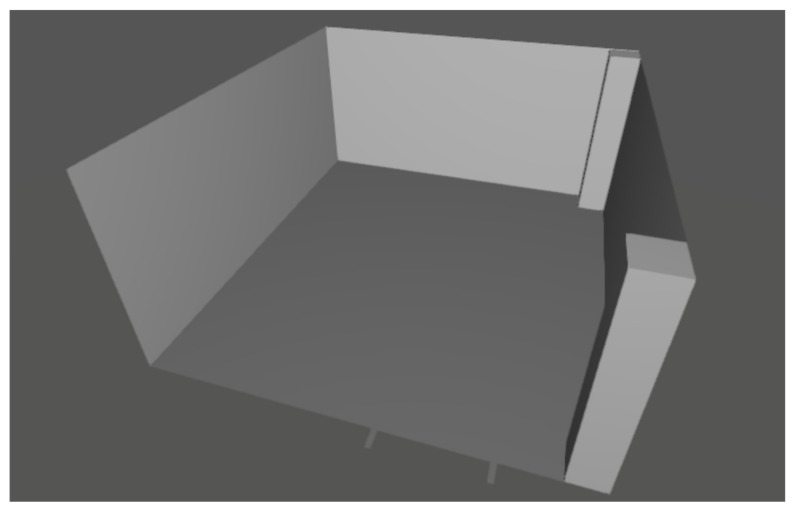
3D model of an empty room.

The design of daylighting of the working environment of the assembly room consists in defining new building openings. In accordance with the sequence of the proposed procedure, the limit values for daylighting are set and controlled in accordance with STN 73 0580-1 [43]. The created space model is shown in Figure 2.

**Figure 3:**
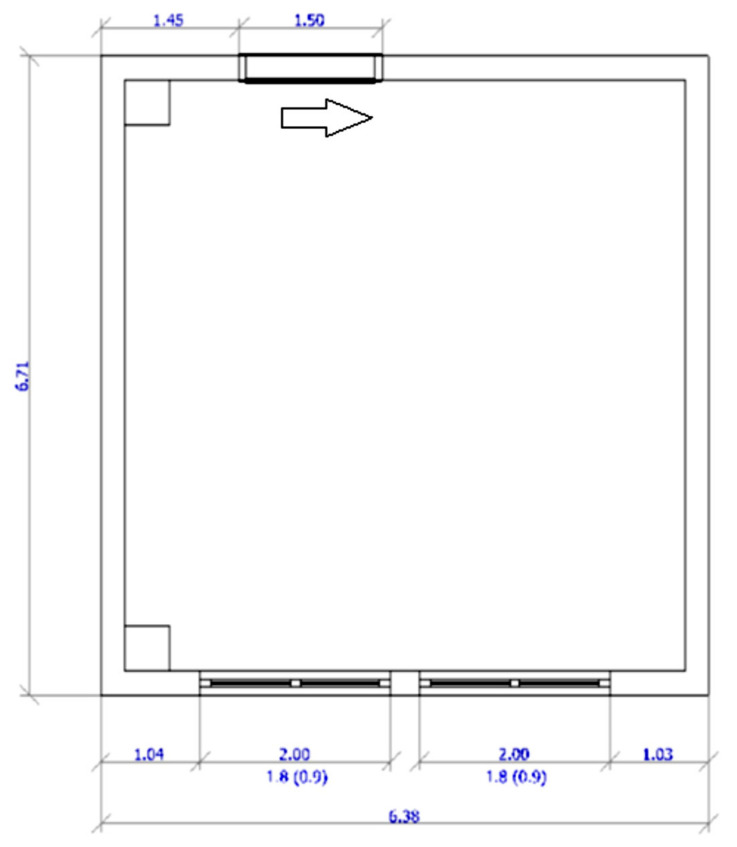
Schematic presentation of new building openings position.

**Figure 4:**
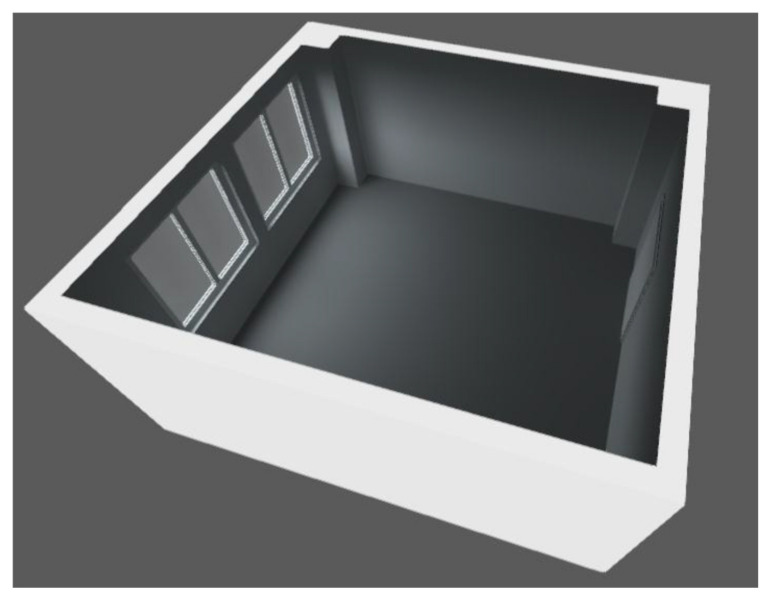
3D model of new building openings position.

The design of the building openings indicated the installation of two windows (Table 1) of 2000 × 1800 mm, with a sill height of 900 mm from the floor plane. In the following figures, the position (in meters) of the individual building openings is precisely defined: windows and sliding doors whose direction of opening is indicated by an arrow (Figure 3). A schematic representation is complemented by a presentation of the compiled 3D model (Figure 4).

Given that the assembly room is situated in an industrial hall, so that it is connected to the outside environment by only one wall, it was necessary to choose larger and wider windows, to ensure at least a minimum required value of daylight factor D = 1.5%. The insulating triple glazing (4-mm clear glass, 18-mm gaps filled with argon) was due to its affordability and application, which is in a non-residential and warehouse space, and to meet the thermal requirements specified in the STN 73 0540-2: 2012 [44] standard. Since this is an assessment of daylighting, by defining the building openings, the design phase of redesigning the daylighting of the working environment of the assembly room was completed.

**Figure 5:**
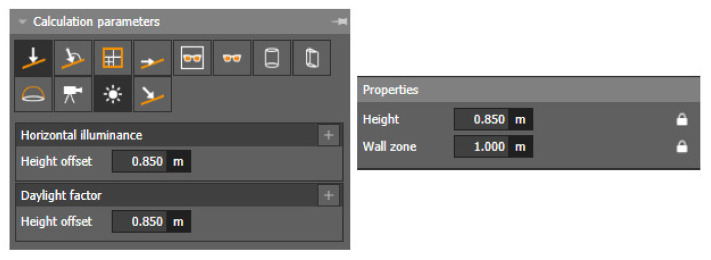
Calculation area settings for daylighting.

Since it is a design of daylighting for a room that does not contain any objects, it is sufficient to create one calculation area that is 1 m from the room edge, according to STN 73 0580-1 [43]. The settings of the individual calculation parameters for the evaluation of the daylight of the assembly room are shown in Figure 5.

For the final evaluation of the new assembly room design, it was also necessary to define the sky model; the sky model was defined according to the standard, cloudy sky. By setting the calculation areas and limit values, the phase of defining the calculation parameters was completed and the simulation itself was carried out.

After the simulation of the daylighting of the assembly room, the overall suitability of the proposed model was assessed from the following aspects: the assessment of the daylight factor, the uniformity of illumination, and the distribution of luminous flux. The achieved numerical results are interpreted in Table 2.

**Figure 6:**
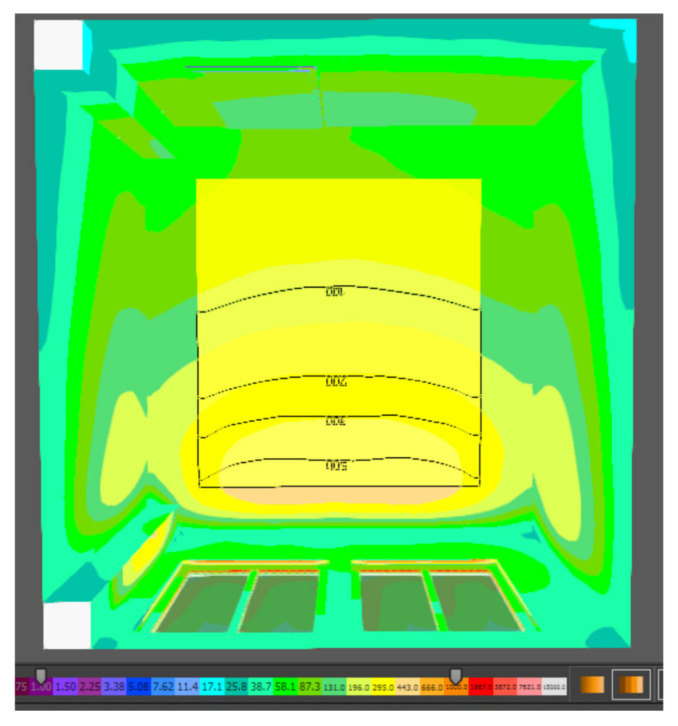
Isophote of daylighting in the assembly room.

Based on the standard and general requirements for the design of daylighting, the assembly room under consideration can be assigned to the IV class of moderate visual activity. For this class, a minimum daylight factor of 1.5 for side lighting is determined. As shown in the table, the average daylight factor for the new design of the assembly room is 1.586%, which is in accordance with the normative requirements. The uniformity of illumination was calculated from the average and minimum value of the illumination intensity achieved on the computational surface. From the results obtained, it can be stated that the proposed solution achieves a uniformity of illumination of 0.30, and thus fulfils the minimum requirement of uniformity of daylight of 0.2. The graphical interpretation of the uniformity of the illumination of the assembly room by means of the isophote display is shown in Figure 6.

Based on the results of the simulation according to the above figure, it can be stated that the luminous flux of daylighting is not evenly distributed; the space is located near the proposed building openings and the space on the other side of the room near the door is the least illuminated. Therefore, when using daylighting in each design, it is recommended that the assembly workplaces be located close to the openings, in order to ensure sufficient daylighting.

### 4.2. Artificial Lighting Design

The design of artificial lighting was based on the constructed model of daylighting. According to the rationalization procedure of work with simulation models, the limit values in accordance with EN 12 464-1 were defined in the next step of the construction phase. Since it is a redesign of artificial lighting after the rationalization procedure, the definition of building openings was omitted, and the lighting system was defined and created. The lighting system consists of two luminaires, the basic specifications of which are given in Table 3.

**Figure 7:**
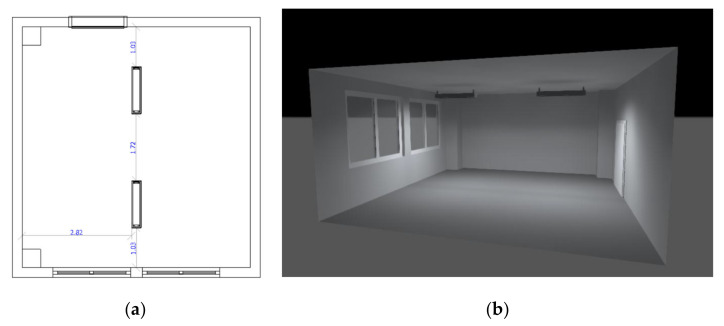
Schematic representation of the location of the artificial lighting system (**a**), and the 3D model of the location of the artificial lighting system (**b**).

The luminaire was chosen for the assembly room to ensure the required illumination intensity and the required chromaticity temperature, which should be approximately 6500 K (daylight level, circadian effect index approaching 100%). It is also necessary to consider the requirement for the color rendering index for a given room type to be higher than 80, when redesigning the lighting as cool day white. Since no type of artificial lighting has been placed so far in the assembly room, it is possible to create a new luminaire arrangement. The arrangement of the lighting system with the indication of the exact positions of the individual luminaires and the 3D visualization is shown in Figure 7.

**Figure 8:**
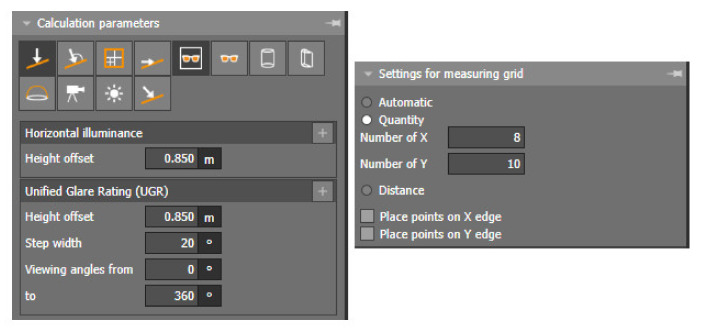
Calculation area settings for artificial lighting.

For the evaluation of the proposed artificial lighting, computational area was used: the plane used in daylighting design. For the overall assessment of the suitability of the proposed model, the calculation parameters illumination intensity and illumination factor UGR are defined for the determined calculation area (Figure 8).

By defining the calculation parameters, the second phase of the redesign was completed: the phase of defining the calculation parameters. After the simulation was carried out, as in the design of daylight, the overall suitability of the proposed model was assessed from the following points of view: illumination intensity, illumination uniformity, and UGR illumination factor. Table 4 provides a numerical overview of the achieved values of the monitored parameters.

**Figure 9:**
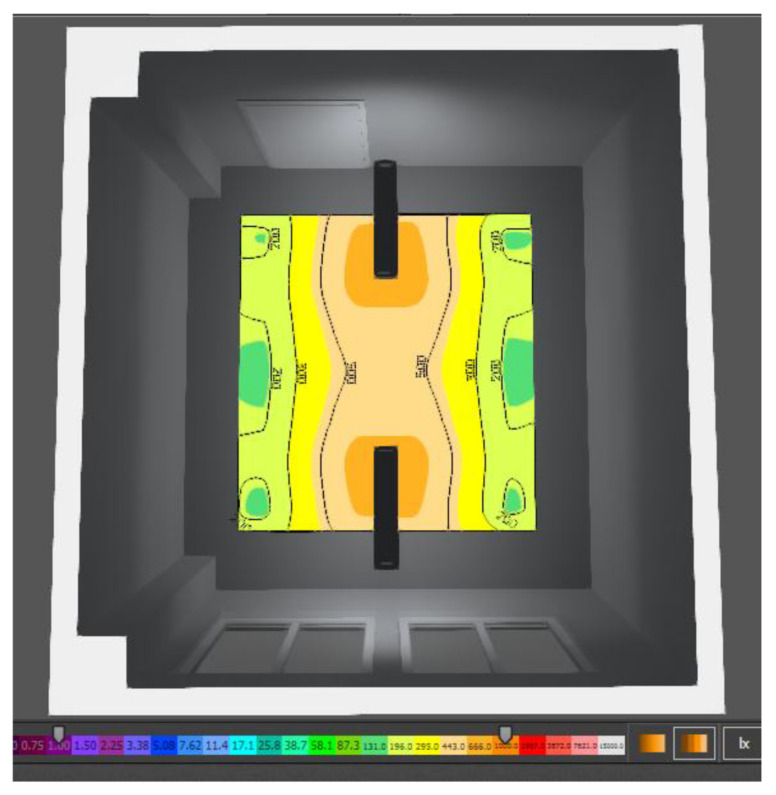
Isophote of artificial lighting in calculating the area.

Since the object under consideration will be used as a room for the execution of assembly work, it is necessary to ensure an average intensity of artificial lighting in the room of a minimum required value of 300 lx. As shown in the summary table of the artificial lighting results of the new design for the assembly room, the average value of the lighting intensity is 423 lx; thus, meeting the minimum requirement for the artificial lighting intensity. The isoforms on the computational plane are shown in Figure 9.

**Figure 10:**
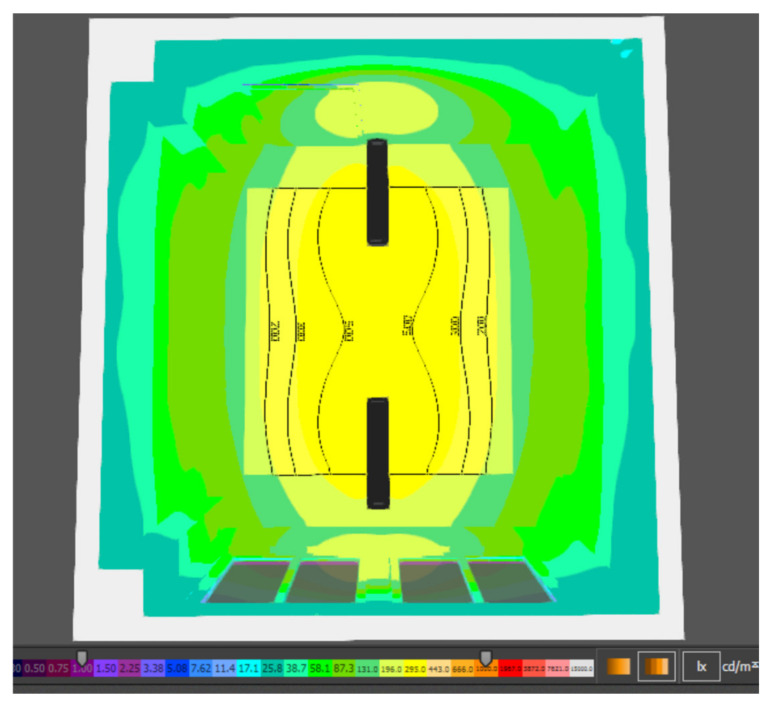
Isophote of artificial lighting in the assembly room.

In the immediately surrounding area, which according to EN 12 464-1 means a 0.5 m wide belt surrounding the workstation, the uniformity of illumination should be 0.5 or more. Given the fact that no precise workplaces are currently defined in the assembly room, the proposed model assessed the uniformity of illumination on the computational surface; a plane that is 0.45 (Figure 10).

**Figure 11:**
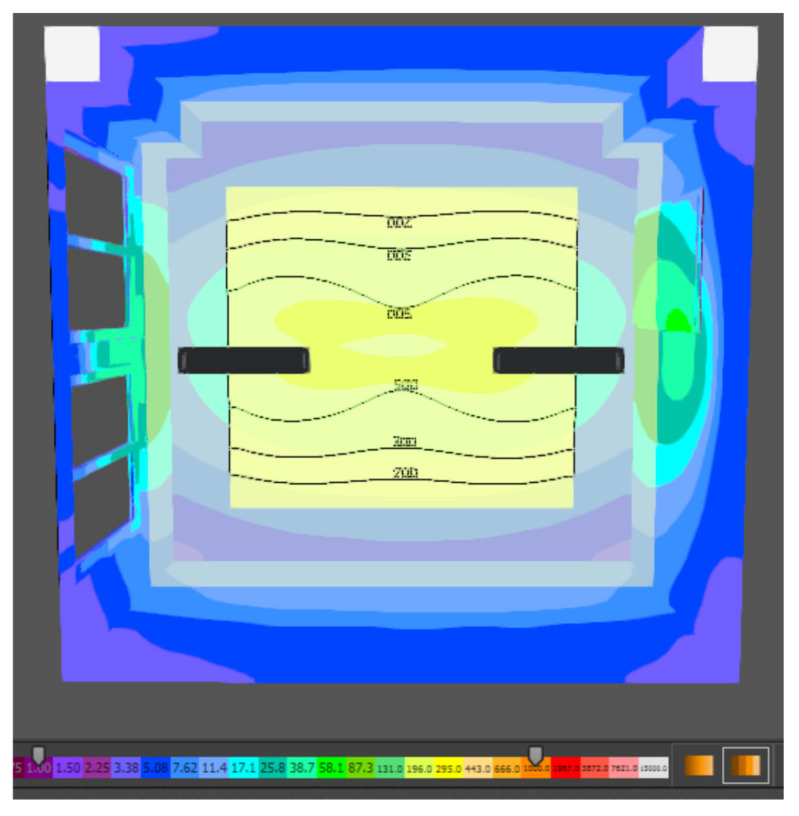
Brightness distribution of artificial lighting in the assembly room.

According to the artificial lighting standard, the brightness is determined by the reflection factors and the surface illumination. The graphical interpretation of the brightness distribution for the evaluated plane is shown in Figure 11.

### 4.3. Mixed Lighting Design

**Figure 12:**
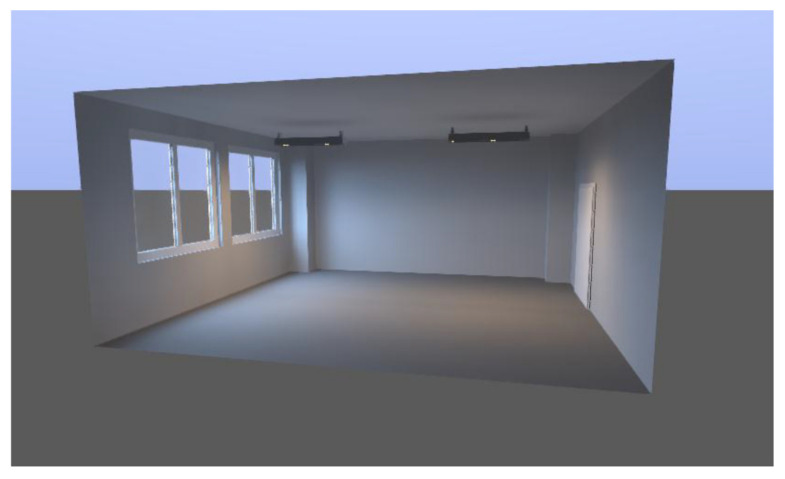
3D model of mixed lighting in the assembly room.

The design of the mixed lighting of the assembly room (Figure 12) working environment is a combination (control) of the design of the daylighting and the artificial lighting of the assembly room mentioned in the previous parts of this article. There is no separate standard for the assessment of mixed lighting, so the assessment is based on the assessment of daylighting and artificial lighting, with a view to controlling the possible occurrence of light overexposure.

**Figure 13:**
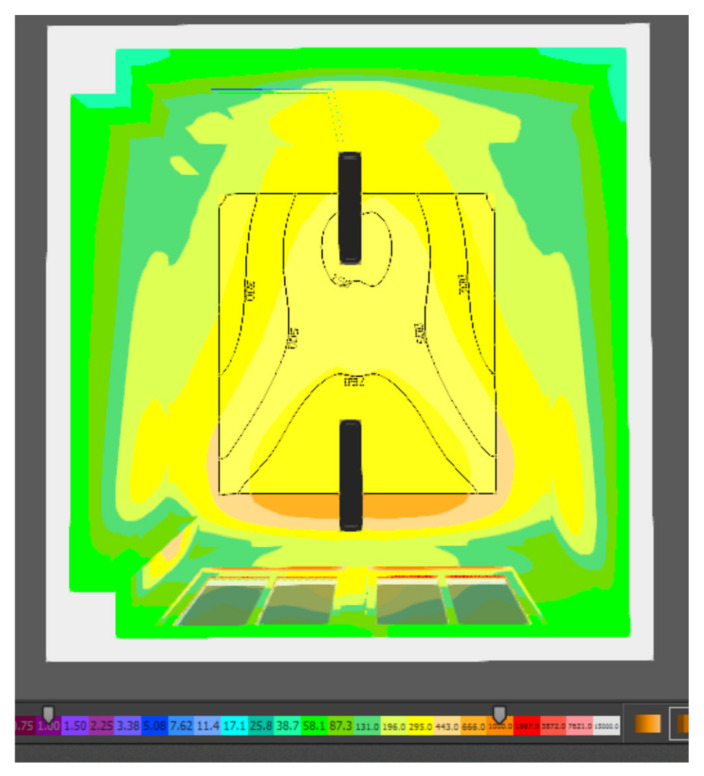
Isophote of mixed lighting.

When assessing the design of the mixed lighting, it is necessary to respect the condition of a minimum value of 300 lx and a daylight factor of at least 1.5% for side lighting. Figure 13 shows a graphical interpretation of the achieved illumination intensity values.

Parameters obtained by simulation confirm the correctness of the assembled models of daylighting and artificial lighting. The average intensity of the mixed lighting is twice as high as the minimum required value, and the UGR value remains unchanged compared to the artificial lighting design. The results of the simulation are shown in Table 5.

The simulation results in the table above show that all monitored parameters are in compliance with the requirements, and it can be stated that the overall design of daylighting, artificial, and mixed lighting for the assembly room conforms to the proposed rationalization procedure work with simulation models, and in terms of legislation and requirements of standards, it is satisfactory and applicable in practice.

## 5. Results and Discussion

**Figure 14:**
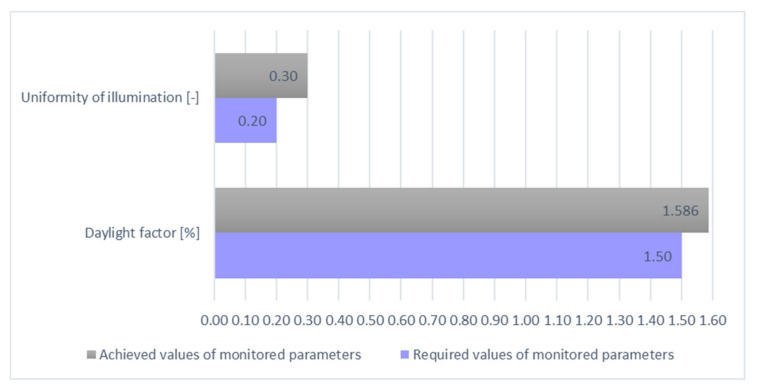
Graphical evaluation of daylighting parameters.

**Figure 15:**
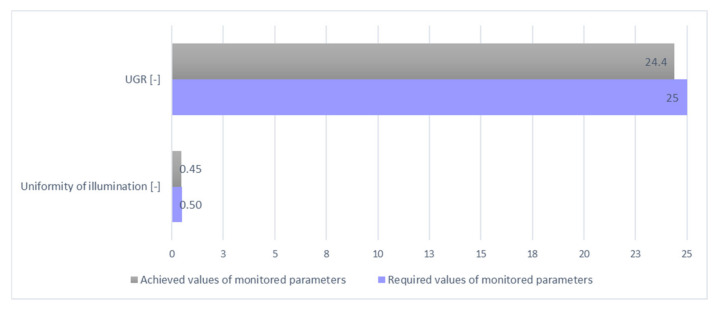
Graphical evaluation of artificial parameters.

**Figure 16:**
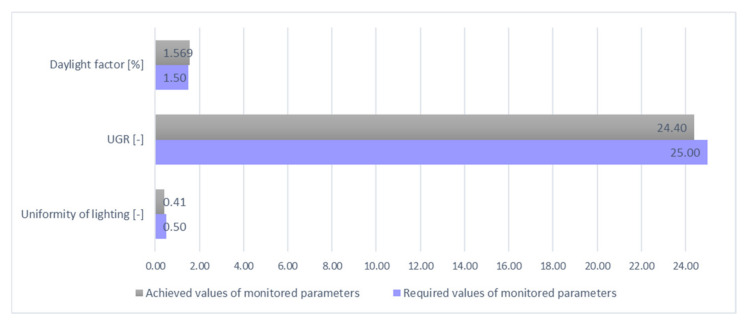
Graphical evaluation of mixed parameters.

In all the proposed solutions, values higher than the values required or in the assessment of the glare of the space were achieved, and values were lower than the maximum allowed. When assessing the uniformity of artificial and mixed lighting, the calculated value appears to be insufficient, but in this case, it is the required value for a particular place for performing a visual task, or a visual task in the immediate proximity. However, the layout of individual workplaces has not yet been determined in the empty room model, and therefore it is necessary to verify this value again in cooperation with the rationalization procedure of the work with simulation models. In view of the results achieved, it is best to locate workplaces from the side around the windows; thus, ensuring the required direction of illumination of the workplace, from the left and from the top. The overall technical evaluation of the lighting of the proposed assembly room model is given in Table 6 and Figure 14, Figure 15 and Figure 16. In the last column of Table 6 are presented the absolute values, as some of the monitored parameters need to be achieved with a higher degree of difference (illumination, daylight factor) and some are better with lower values (uniformity, UGR) as required values.

Based on the specific values found in the previous table and figures, it can be stated that the overall design of the daylighting, artificial, and mixed lighting of the assembly room is satisfactory and can be applied directly in practice, after determining the specific workplaces and a subsequent inspection.

## 6. Conclusions

Nowadays, industries are increasingly marked by digital technologies. The manufacturing industry is no exception to this. It is the manufacturing industry that is the industry that has seen an increase in the use of digital technology in recent years, with the primary goal of achieving a dynamic manufacturing environment, with the ability to develop continuous innovations in processes or products. This paper presents a procedure on how to work with the available digital tools. The main aspect of this work lies in a strict sequence of steps for the redesigning of lighting intended for production and industrial halls. General design solutions for lighting are based on documents for lighting projects in houses, apartments, or offices. This sequence addresses the issue directly, with a focus on production halls and industrial plants. The other application is to verify the new practical research in the residential lighting (for example). It describes the proposed general rationalization procedure of work with simulation models in the area of evaluation of the physical factors of the working environment; lighting. The proposed procedure was verified on a practical solution to the redesign of a assembly room, in which daytime, artificial, and mixed lighting was designed. Based on the research and subsequent creation of a proposal for a rationalization procedure for work with simulation models, the following benefits for the scientific field and practice were determined:

- Proposal of a methodology for working with lighting technical simulation tools.
- Utilization of the proposed procedure for the realization of the redesign of working environment lighting.
- Enhancing the knowledge of computer support in ergonomics.
- The practical and realistic design of the redesign of assembly room lighting.

With the help of a rationalization procedure of work with simulation tools, modification of the assembly room was designed, which meets all the necessary legislative requirements and requirements set by standards. The present article provides the sequence of work, with a simulation model in ergonomics, and the possibility to use the tools of a digital enterprise not only in areas such as production or planning, but also in the areas of assessment of the working environment namely in the assessment of daylighting, artificial lighting, and mixed lighting. The evaluation of the physical factors of the working environment is a very important issue in the assessment process of public health quality in the workplace. In any case, if the analyzed work environment and its factors are incorrectly assessed, whether due to an incorrectly designed model, non-compliance with sequences of work with the model, or bad interpretation of results, such a misjudgment can have a very negative impact on occupational safety and health of workers. This article provides new approaches and a methodology for this form of ergonomic rationalization, bringing it up to date. By applying this sequence, it is possible to ensure a correct design procedure, which also ensures quality management and eliminates the subsequent technical shortcomings that are closely linked to the economy of the company. The presented proposal can be extended in the future by research focused on the assessment of economic impacts and management processes, with consequent results in the assessment of possible emerging risks associated with the issue.

## Figures and Tables

**Table 1:** Technical specification of proposed building openings.

Number of Chambers	5
Construction depth (mm)	70
Window fittings	Sigenia-Aubi Titan AF with an anticorrosive coat
Type of glazing	triple pane insulated glass 4/18/4/18/4 (Ag)
Thermal transmittance of glass U_g_ (W/m^2^ K)	0.7
Thermal transmittance of frame U_f_ (W/m^2^ K)	1.2
Thermal transmittance of indeed the whole window U_w_ (W/m^2^ K)	0.95
Material and colour of frame	double-sided white plastic
Windows reflection (%)	14.8
Refractive index	1.5

**Table 2:** The new design of the assembly room: results of daylighting simulation.

Monitored Parameters	Achieved Values of Monitored Parameters
Daylight factor (%)	1.586
Uniformity of illumination (–)	0.30

**Table 3:** Technical specification of the proposed artificial lighting.

Efficiency (%)	86.88
Light flux of light (lm)	6646
Power (W)	118
Colour rendering index (Ra)	85
The chromaticity of light (K)	6500

**Table 4:** The new design of the assembly room, results of the artificial lighting simulation.

Monitored Parameters	Achieved Values of Monitored Parameters
Average illumination intensity (lx)	423
Minimum illumination intensity (lx)	189
Maximum illumination intensity (lx)	729
Uniformity of illumination (–)	0.45
UGR (–)	24.4

**Table 5:** The new design of the assembly room, results of the artificial lighting simulation.

Monitored Parameter	Achieved Values of Monitored Parameters
Average illumination intensity (lx)	619
Minimum illumination intensity (lx)	253
Maximum illumination intensity (lx)	1217
Uniformity of illumination (–)	0.41
UGR (–)	24.4
Daylight factor (%)	1.569

**Table 6:** Technical evaluation of lighting design.

Illumination Type	Monitored Parameters	Achieved Values of Monitored Parameters	Required Values of Monitored Parameters	Difference between Achieved and Required Values (Absolute Value)
daylight	Daylight factor (%)	1.586	1.5	0.086
	Uniformity of illumination (–)	0.30	0.2	0.1
artificial	Average illumination intensity (lx)	423	300	123
	Uniformity of illumination (–)	0.45	0.5	0.05
	UGR (–)	24.4	25	0.6
mixed	Average illumination intensity (lx)	619	300	319
	Uniformity of lighting (–)	0.41	0.5	0.09
	UGR (–)	24.4	25	0.6
	Daylight factor (%)	1.569	1.5	0.069

## Data Availability

Not applicable.
